# Supplementary material for: Transcriptomic Landscape of Circulating Extracellular Vesicles in Heart Transplant Ischemia–Reperfusion
Source: Genes (Basel). 2023 Nov 18;14(11):2101. doi: 10.3390/genes14112101 (PMC10671425; doi:10.3390/genes14112101)
Supplement: Supplementary file 1 [file genes-14-02101-s001.zip › SupplementaryFigures_final_v2.pdf]

Supplementary Figures

Transcriptomic Landscape of Circulating Extracellular Vesicles in Heart Transplant Ischemia-Reperfusion

SeoJeong Joo MSc [1], Kishor Dhaygude PhD [1], Sofie Westerberg BM [1],

Rainer Krebs PhD [1], Maija Puhka PhD [2], Emil Holmström MD [1],

Simo Syrjälä MDPhD[1,3], Antti Nykänen MD PhD[1,3], and Karl Lemström MD PhD[1,3]

1 Transplantation Laboratory and Translational Immunology Research Program, University of Helsinki, Helsinki, Finland

2 Institute for Molecular Medicine Finland FIMM, EV and HiPREP core, University of Helsinki, Helsinki, Finland

3 Heart and Lung Center, Helsinki University Hospital, University of Helsinki, Helsinki, Finland

*** Correspondence: karl.lemstrom@helsinki.fi**

Table of Contents

Figure S1. Comparison of enriched biological terms for differentially expressed genes (DEGs) and high-power discrimination genes (HPDs) 2

Figure S2. Scatter plots of REVIGO-based summarized gene ontology terms using differentially expressed genes found in 12-hour EV samples when compared to 0 h 3

Figure S3. Heart-specific cell signatures related to upregulated and downregulated DEG at 12 hours after reperfusion 4

Figure S4. Biological functions and pathways enriched by statistically significant genes that are associated with primary graft dysfunction after heart transplantation 5

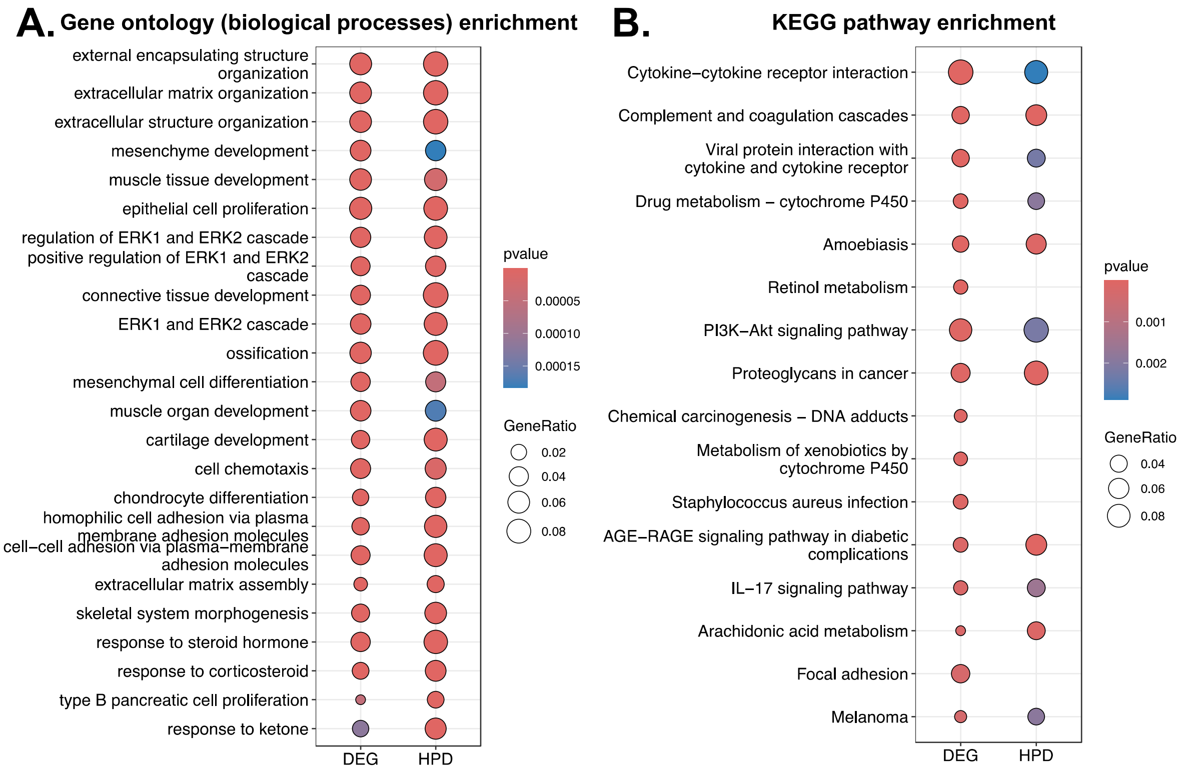

Figure S1. Comparison of enriched biological terms for differentially expressed genes (DEGs) and high-power discrimination genes (HPDs)

To compare the biological characteristics between the overall differentially expressed genes (DEGs) (*n*=1317) and the high-power discrimination genes (HPDs) showing sensitivity and specificity of 80% (*n*=357), we performed analyses to enrich relevant biological annotations. The results are presented as **(A)** a dot plot illustrating enriched gene ontology terms related to biological processes, and **(B)** another dot plot illustrating enriched KEGG pathway terms.

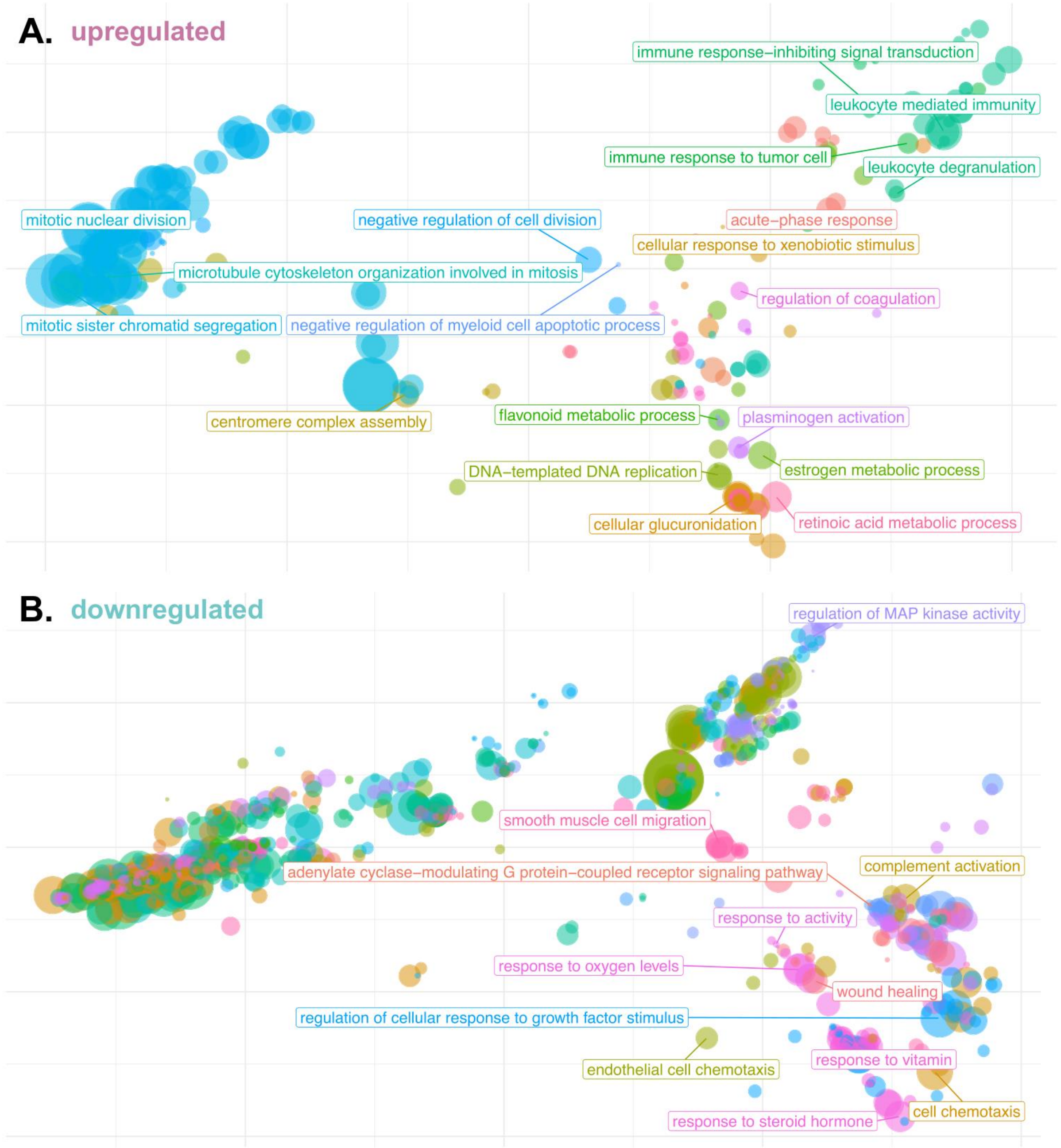

Figure S2. Scatter plots of REVIGO-based summarized gene ontology terms using differentially expressed genes found in 12-hour EV samples when compared to 0 h RRVGO package in R was used to calculate semantic similarity scores (Resnik's and Lin's method [21]) for enriched terms in the original gene ontology enrichment analysis. The scatterplots illustrate the similarity among enriched terms by distance, and the size of each bubble represents the size of the correspondent gene set. Based on the semantic similarity scores, the plot illustrates **(A)** enriched gene ontology terms of the upregulated genes. and **(B)** enriched gene ontology terms of the downregulated genes.

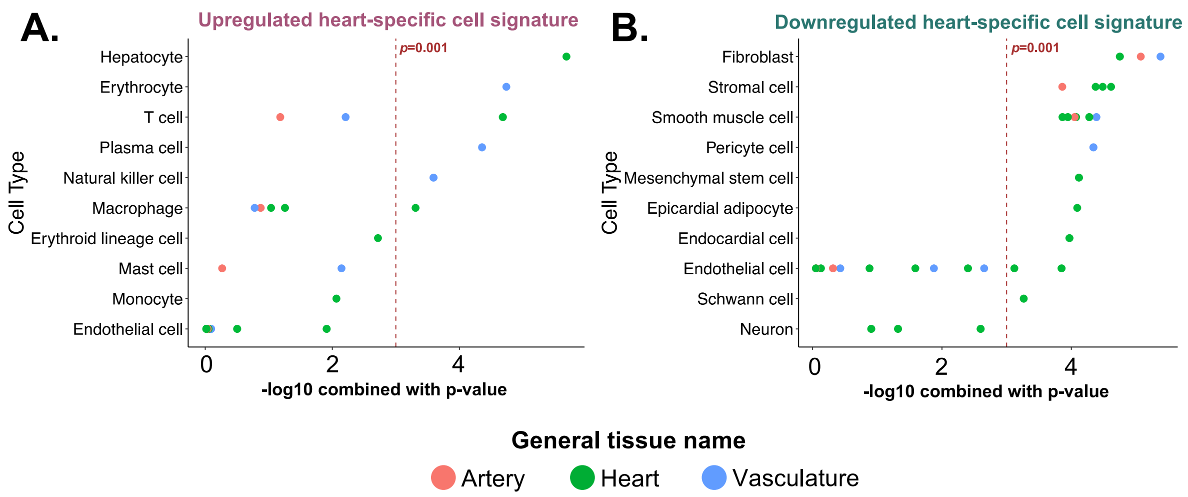

Figure S3. Heart-specific cell signatures related to upregulated and downregulated DEG at 12 hours after reperfusion

To gain detailed insight into heart-specific cell signatures, we conducted WebCSEA analysis separately for **(A)** upregulated DEGs and **(B)** downregulated DEGs.

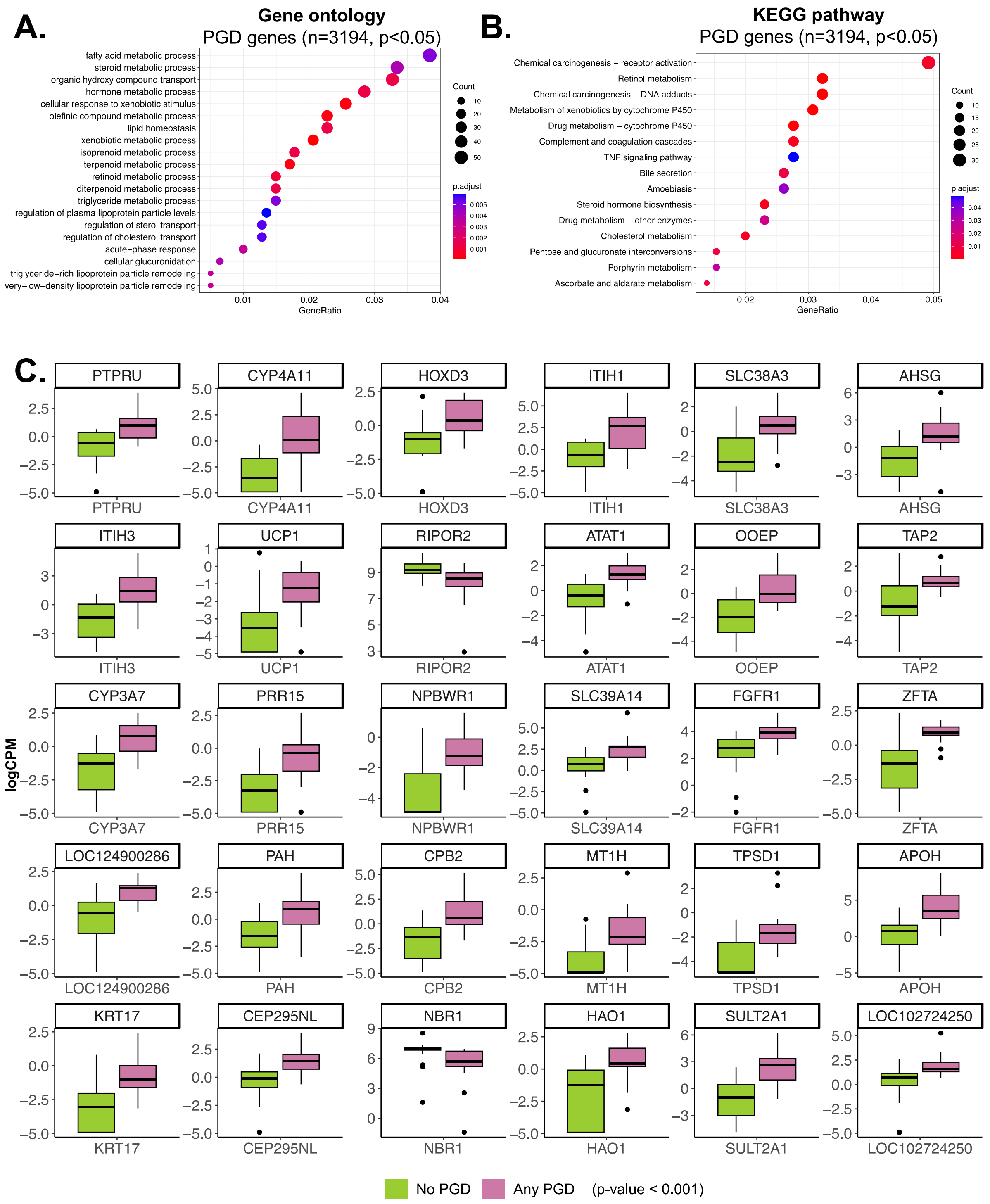

Figure S4. Biological functions and pathways enriched by statistically significant genes that are associated with primary graft dysfunction after heart transplantation 3194 genes were relevant to the occurrence of primary graft dysfunction after heart transplantation (p<0.05). The dot plots illustrate **(A):** enriched gene ontology terms for biological processes and **(B):** relevant KEGG pathways using the 3194 primary graft dysfunction-related genes. **(C):** Furthermore, we found 30 protein-coding genes that possibly have stronger correlations with primary graft dysfunction after heart transplantation, by implementing a stricter p-value cut-off (p<0.001).
